# Exploring Determinants of Compassionate Cancer Care in Older Adults Using Fuzzy Cognitive Mapping

**DOI:** 10.3390/curroncol32080465

**Published:** 2025-08-16

**Authors:** Dominique Tremblay, Chiara Russo, Catherine Terret, Catherine Prady, Sonia Joannette, Sylvie Lessard, Susan Usher, Émilie Pretet-Flamand, Christelle Galvez, Élisa Gélinas-Phaneuf, Julien Terrier, Nathalie Moreau

**Affiliations:** 1 Faculty of Medicine and Health Sciences, Université de Sherbrooke, Longueuil, QC J4K 0A8, Canada; catherine.prady.med@ssss.gouv.qc.ca; 2 Centre de Recherche Charles-Le Moyne, Longueuil, QC J4K 0A8, Canada; sylvie.lessard@usherbrooke.ca (S.L.); susan.elizabeth.usher@usherbrooke.ca (S.U.); 3 Centre Léon Bérard, 69008 Lyon, France; chiara.russo@lyon.unicancer.fr (C.R.); catherine.terret@lyon.unicancer.fr (C.T.); emilie.pretet@lyon.unicancer.fr (É.P.-F.); christelle.galvez@lyon.unicancer.fr (C.G.); 4 Centre Intégré de Santé et de Services Sociaux de la Montérégie-Centre (CISSSMC), Greenfield Park, QC J4V 2G9, Canada; sonia.joannette@usherbrooke.ca (S.J.); elisa.gelinas-phaneuf.cisssmc16@ssss.gouv.qc.ca (É.G.-P.); julien.terrier.cisssmc16@ssss.gouv.qc.ca (J.T.); nathalie.moreau.cisssmc16@ssss.gouv.qc.ca (N.M.)

**Keywords:** geriatric oncology, older adults, quality improvement, deliberation, fuzzy cognitive mapping

## Abstract

The growing number of older adults with cancer confront practical and organizational limitations that hinder their ability to obtain care that is adapted to their health status, needs, expectations, and life choices. The integration into practice of evidence-based and institutional recommendations for a geriatric approach and person-centered high-quality care remains incomplete. This study uses an action research design to explore stakeholders’ perspectives of the challenges involved in translating the established care priorities into a compassionate geriatric approach in oncology and identify promising pathways to improvement. Fifty-three stakeholders participated in focus groups to create cognitive maps representing perceived relationships between concepts related to compassionate care of older adults with cancer. Combining maps results in a single model constructed in Mental Modeler software to weigh relationships and calculate concept centrality (importance in the model). The model represents stakeholders’ collective perspective of the determinants of compassionate care that need to be addressed at different decision-making levels. The results reveal pathways to improvement at systemic, organizational, practice, and societal levels. These include connecting policies on ageing and national cancer programs, addressing fragmented care through interdisciplinary teamwork, promoting person-centered care, cultivating relational proximity, and combatting ageism. Translating evidence-based practices and priority orientations into compassionate care rests on collective capacities across multiple providers to address the whole person and their unique trajectory.

## Simple Summary

Cancer can develop at any age, but its incidence rises dramatically in later life. Research points to persistent shortcomings in the care of older adults with cancer despite internationally recognized priority practices. The current study explores the complexity of implementing a geriatric approach in oncology grounded in a foundation of compassionate care. Compassion requires that healthcare providers strive to proactively alleviate another person’s suffering as they identify pathways to high-quality and safe care. We engage stakeholders from different perspectives—decision-makers, researchers, and providers and users of cancer care—in a systematic cognitive mapping exercise to clarify the contributors and impediments to progress. The results show that it is possible for stakeholders with potentially competing perspectives to arrive at a common understanding of the prerequisites to enacting compassionate care for older adults with cancer. We find strong consensus on the need to integrate a focus on older adults in national cancer programs, address fragmented care through interdisciplinary teamwork, promote person-centered care through relational proximity, and combat ageism. Translating evidence-based practices and priority orientations into compassionate care rests on collective capacities across multiple providers to address the whole person and their unique trajectory.

## 1. Introduction

Cancer can develop at any age, but its incidence rises dramatically in later life due to an accumulation of risk factors and less effective cellular repair mechanisms [1]. Worldwide population ageing will inevitably result in more older people being affected by cancer requiring care and poses challenges to the existing practice. A geriatric approach in oncology is recommended for people over 70 or 75 years old given the progressive decline in physiological function related to age and the physical and psychological impact of cancer and its treatment [2]. While the value of a geriatric approach in oncology has been recognized for over 25 years, it is still incompletely and unevenly integrated into practice [3].

Research points to many shortcomings in care delivery. Uncertainties regarding whether to screen for cancer [4], longer delays for diagnosis [5], underrepresentation in clinical research and a lack of trials designed specifically for older frail adults [6], concerns about under- or over-treatment [7], and a failure to meet whole-person support needs [8] remain too common [3,8,9]. Additionally, thresholds based on chronological age poorly reflect the heterogeneity of older adults in terms of comorbidity; cognitive, nutritional, and functional status; life situation; and expectations, all of which affect appropriate care [2]. The literature shows that practical and organizational limitations hinder the ability to tailor care to the health conditions, needs, expectations, and life choices of older adults [3,10,11,12].

An integrated geriatric approach in oncology is supported by evidence [13] and worldwide institutions [14,15,16,17,18]. The publication in 2011 of the International Society of Geriatric Oncology (SIOG)’s first set of priorities for the improvement of care for older adults with cancer sparked an interest in research, training, and addressing unmet needs [19]. In 2021, the SIOG expert panel updated this work and released 12 strategic priorities for moving ahead on high-quality equitable geriatric oncology care [14]. These priorities are grouped around four main themes: professional training and public education; coordinated care models of clinical practice; research, including an evaluation of the benefits of personalized medicine; and collaboration to achieve excellence supported by appropriate funding. Integrating these priorities into practice would contribute to a more tailored and equitable cancer system.

However, patient experience [20] of compassionate care, while recognized as a core component of high-quality cancer care [21], remains under-addressed in these priorities. The seminal work by Sinclair and colleagues [22,23] considers compassion as a distinct and overarching component of quality care: it requires “healthcare providers to invoke their personhood along with their clinical proficiencies to have an optimal effect in alleviating patient suffering” [22] (p. 202). A recent literature review reports a growing consensus that compassion is relational, consisting of acknowledging, engaging, and proactively attending to another person’s suffering [20]. For a geriatric approach in oncology to be person-centered, a foundation of compassion is essential [24]. Notably, patient experience includes older adults living with and beyond cancer and their lay caregivers in the following text.

It is therefore imperative to better articulate the mental models around compassionate care in geriatric oncology and the factors underlying the gap between scientific, practical, and experiential knowledge to collectively arrive at realistic and mutually acceptable strategies for improvement on priority areas. Compassionate care involves listening, understanding, accepting, and accompanying patients in therapeutic decisions that respond to their individual priorities [20]. It helps alleviate concerns and suffering arising from healthcare systems ill-adapted or ill-equipped to provide whole-person care [25,26].

Translating priorities into compassionate care requires that multiple stakeholders become more informed about the knowledge–practice gap and engage in joint action to improve the health and well-being of older adults with cancer [3]. However, this crucial translation process must contend with relational challenges, and organizational and contextual constraints [27]. A recent literature review concludes that network configurations are important to enabling professionals to act on compassion when providing care [20]. This suggests that compassionate care depends on collective capacity to redesign integrated health networks where specialized geriatric and oncology care, primary care, and community services activate communication mechanisms and collaborate on value-added practices. To face these challenges, cancer care settings are encouraged to integrate the experiential and practical knowledge of multiple stakeholders in seeking solutions that enable compassionate care for older adults with cancer [3,28,29].

Enabling compassionate care is both person and context dependent. The PRISM (Practical Robust Implementation and Sustainability Model) is a context-based implementation science framework, providing a preliminary step as an extension of RE-AIM (Reach, Effectiveness, Adoption, Implementation, Maintenance) [30]. The PRISM aligns with the pragmatic aims of the present study to support the enactment of compassionate care within the consensus priority actions for a geriatric approach in oncology [30]. It highlights the importance of assessing the perspectives of diverse partners to fully understand the contextual characteristics that determine sustainable implementation [31]. The extended framework incorporates the perspectives of people at different decision-making levels to help understand what factors need to be considered and addressed for a successful translation of evidence-based interventions. It also provides a starting point for integrating local experience and context into the enactment of compassionate care and enabling the co-creation of outcomes (RE-AIM).

The current study explores the complexity of implementing compassionate care in geriatric oncology practice with stakeholders from different perspectives to identify promising pathways to improvement. The interrelated objectives are as follows: (1) to determine the relationships between the critical factors involved in integrating a compassionate geriatric approach in real-world oncology practice, (2) to elicit different stakeholder views on the positive or negative influence of various determining factors, and (3) to draw up realistic priority orientations and actions for improvement.

## 2. Materials and Methods

### 2.1. Study Design

An action research design [32] is chosen to be consistent with our intention to enable stakeholders to think beyond current limitations in the healthcare system and consider how barriers to acting on compassionate care could be overcome in geriatric oncology. The approach is rooted in “the science of solutions” [33,34] that brings together researchers and actors from the field affected by the challenges of implementing compassionate geriatric care in oncology. This approach facilitates trust-building and open exchange between people with different mental models based on scientific, practical, and experiential knowledge. When exploring questions of values and objectives, and emotionally-charged issues such as compassionate care which are subject to competing interpretations, trust-building approaches help safeguard the relevance of research questions, the data collected, and interpretations [35]. We employ fuzzy cognitive mapping (FCM) to structure the study process and develop a pragmatic understanding of the complex dynamics revealed in stakeholder deliberation. This choice is supported by the recognition of FCM as a rigorous and transparent approach to integrating theory and real-world perspectives, extracting meaning, and guiding decision-making [36,37,38]. FCM is especially useful when dealing with multidimensional concepts such as compassionate care that are not easily measurable and have multiple definitions [39].

### 2.2. Participants

Fifty-three participants took part in a one-day deliberative symposium to explore the concepts involved in compassionate care for older adults with cancer from multiple perspectives. The in-person symposium, held in October 2024, included formal presentations on cancer care delivery (researcher), national cancer programs (policymaker), international priorities (SIOG past president), and the models of care in geriatric oncology (geriatrician, the leader of a geriatric oncology clinic), and a roundtable discussion among people with lived experience of providing or receiving cancer care. These sessions provided opportunities to exchange insights into the current context of geriatric oncology and clarify the definitions of concepts to be used in FCM deliberations. The reflective FCM deliberation [40] then engaged the participants involved in researching, practicing, and using cancer care and services for older adults in France and Québec. Reflective thinking in multi-stakeholder deliberation offers an opportunity to examine a situation while heightening awareness of one’s own beliefs, values, and practices. It allows people to learn from their own experiences and from new knowledge and integrate this learning into their efforts to confront challenges and improve care outcomes [5,35,41].

Non-probabilistic sampling was used to select knowledgeable participants involved in cancer care who could provide perspectives regarding both evidence and real-world practice [42]. The inclusion criteria were as follows: age ≥ 18 years; experience as one of the following: an older adult with cancer, a lay caregiver, a researcher, a health professional (e.g., allied health professionals, oncologists, geriatricians, primary care providers), a representative of a nonprofit community organization, a decision-maker responsible for governance (e.g., policymakers, managers, municipal leaders); a willingness to share their perspective on compassionate care for older adults with cancer; and the ability to understand and speak French. These eligibility criteria align with the action research objectives of obtaining a wide range of perspectives on the phenomenon of interest [43,44]. Table 1 describes the characteristics of the FCM participants.

### 2.3. Fuzzy Cognitive Mapping Procedure

We explore challenges in the mental models of compassionate care tailored to older adults, such as heterogeneity in their health condition, needs, values, and preferences; chronological vs biological age; and a healthcare system under pressure. We also explore concepts of coordination among medical specialties and between oncology and primary care. Each of these challenge areas is vital to achieving compassionate care, and each is influenced by differences in how they are understood by stakeholders with different knowledge, experience, and values. The intermingled causes and effects between concepts are illustrated during mapping [38,39]. FCM provides a systematic way to raise, contrast, and combine multiple and sometimes competing mental models. It supports the analysis of complex problems that often involve choosing between plausible solutions in a context of uncertainty [39]. FCM is a constructive methodology that involves aggregating a wide range of points of view on an issue held by different stakeholders illustrated by quantified relationships on a visual support [37,45].

**Figure 1:**
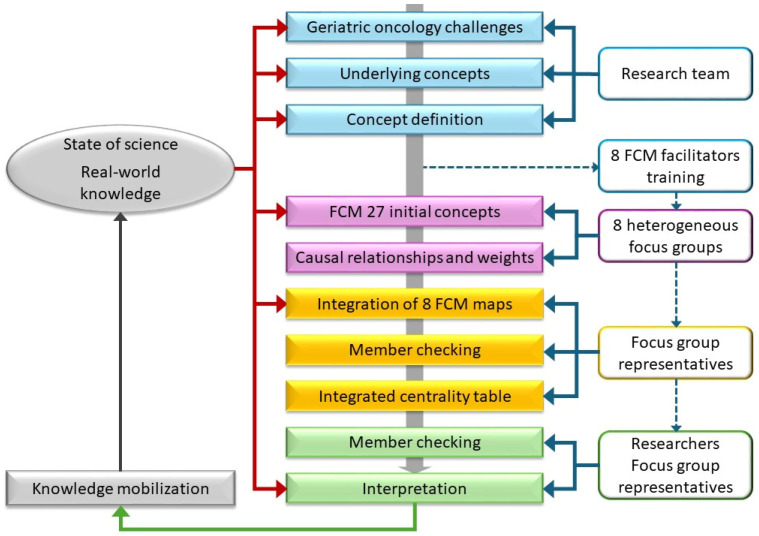
Fuzzy cognitive mapping data process. Colors are used to categorize the key steps: preparation (blue), data collection during the FCM session (pink), analysis (yellow), and interpretation (green). Dotted arrows indicate participant contributions. FCM: fuzzy cognitive mapping.

The established FCM methodologies were adapted to provide a systematic, but not necessarily linear process of providing meaningful information for various stakeholders in cancer systems (Figure 1) [37,38,43,46]. First, geriatric oncology challenges and the concepts underlying them were defined by drawing on research evidence from SIOG priorities and research team member expertise (see the lexicon in Supplementary Table S1: Definitions of concepts). Next, qualitative and semi-quantitative data were collected sequentially on the causal relationships between concepts. The emphasis of the qualitative data was on the characteristics of discourse used to describe participants’ mental models during the FCM-related discussions [38]; the semi-quantitative data (weight of relationships) explored indicative “what if” rather than predictive statistics to determine the relationships between concepts [47]. Data collection, analysis, and interpretation were undertaken to reflect the FCM objectives of making sense of concepts for real-world practice and mobilizing knowledge. The mapping resulted in a visual network map where boxes represent the concepts involved in compassionate geriatric oncology joined by arrows representing positive, negative, or neutral influence. This basic structure, displayed on a whiteboard, enabled participants to assemble their multiple perspectives on the causal relationships between concepts [47].

#### 2.3.1. Preparation

The research team explored the recent literature related to compassionate geriatric oncology and practice guidelines from international institutions to review the state of science. The most frequent concepts were retained from a full text analysis of empirical studies, and literature reviews related to SIOG priorities. Up to 27 underlying concepts (see the lexicon in Supplementary Table S1: Definitions of concepts) were identified and grouped into four main challenges for improving geriatric oncology: variation in detection tools and comprehensive geriatric assessment utilization [3,48], healthcare system fragmentation that impedes coordination and continuity of care [12,13,49,50,51], personalizing treatment according to age or age-related frailties [2], and the complex and uncertain context of care delivery that works against compassionate care for older adults with cancer [3]. Ahead of the symposium, a training session was provided to the facilitators who had previous experience in cancer care and conducting focus groups. The training objectives were as follows: (1) to explain the aim and objectives of multi-stakeholder deliberation based on our previous work [52], (2) to review the ethics information and the consent form [53], (3) to learn how to direct and weigh causal relationships during FCM sessions [45], and (4) to review focus group best practices and time management, notably to address power differentials and ensure equitable speaking time among participants [54]. Geriatric oncology challenges and the definitions of underlying concepts, national and international priorities, and real-world experiences were presented and discussed prior to the FCM session.

#### 2.3.2. Data Collection During the FCM Session

Data collection sought to explore the influence (positive or negative) of concepts on compassionate care of older adults with cancer and the strength of the causal relationships between these concepts. Participants (*n* = 53) were assigned to eight heterogeneous focus groups [42] of 6 to 8 people in order to capture their various perspectives [55,56]. Each group was provided with a whiteboard displaying compassionate care in the center and the related concepts on the periphery. The group sessions lasted 90 min. Facilitators presented the study objectives and then asked participants to consider the following questions: When thinking about compassionate care for older adults with cancer, how do you think these concepts provide a basis for deepening our understanding of drivers? Which of the peripheral concepts are directly related to the core concept? Are there relationships between the peripheral concepts? Can you draw an arrow to describe the direction of the relationship between concepts? How strong is each of these relationships, between −1 and +1 (−1 = strongest negative relationship; +1 = strongest positive relationship)? What is the key learning arising from your deliberation that you would like to share with decision-makers? The maps of each group were photographically recorded and notetakers captured illustrative quotes during the FCM sessions.

#### 2.3.3. Analysis

Following the work of Sarmiento and colleagues [39], graphic displays of each map including the strength of the relationships between concepts (−1 to +1) were translated into a Microsoft Excel data table. The value in a cell indicated the weight of the influence of the row concept on the column concept. A simple average weight of the relationships across maps was used to combine the maps with equivalent perspectives and relevance [57]. If the weight or direction of influence differed between maps, a member checking process [58] was used to reach a consensus among research team members and participant representatives. Member checking was undertaken the day after the FCM session to obtain a consensus and validate a qualitative summary of the deliberations recorded in writing by notetakers. After initial member checking, the weight values of the concept relationships were entered into Mental Modeler [46] to create a fuzzy cognitive “metamodel” [44] from the aggregation of the eight focus groups. Mental Modeler is a free online modeling tool available to create collective fuzzy cognitive maps; it was used to calculate the centrality of concepts related to compassionate care for older adults with cancer. Centrality (C) is a quantitative measure of the importance of a concept, arrived at by adding up the absolute weight values of incoming and outgoing arrows to identify the concepts that contribute mostly as causes (indegree) or as outcomes (outdegree) [39]. Centrality is always a positive value, but participants described the relationships as negative or positive. Another member checking session was undertaken one month after data collection to validate the metamodel and collectively interpret qualitative and semi-quantitative data. Illustrative quotes retained during member checking were translated from French to English by a co-author (SU) who is bilingual with English as her mother tongue. The relevance of each peripheral concept was then analyzed and interpreted using a clustering approach, taking into account its centrality value and its perceived positive or negative influence on compassionate care for older adults with cancer.

This study was approved by the Research Ethics Board of the Montérégie-Centre Integrated Health and Social Service Centre (2025-940).

## 3. Results

The results present the central role of compassionate care of older adults in oncology and the relationships and influences between compassionate care of older adults with cancer and the 27 predetermined concepts. As expected, given that it was the focus of this study, compassionate care had the highest centrality value (C = 19.71). The quantitative centrality values provided a hierarchical order of the determinants of compassionate care supported by illustrative quotes from the multi-stakeholder deliberation. The qualitative discourse contributed to eliciting priority orientations and actions for improvement.

### 3.1. Compassionate Care as a Core Concept for Improvement

The cognitive maps from the eight multi-stakeholder deliberative groups were aggregated into a metamodel composed of interconnected concepts and the multidirectional relationships between them. The metamodel output figure from Mental Modeler is presented in Supplementary Figure S1: Metamodel aggregating maps from eight focus groups. The figure illustrates the entanglement of relationships and provides a basis for deepening our understanding of where efforts could be made. The participants collectively agreed that compassionate care was a key “to pave the way to integrated geriatric oncology and move forward to effective and safe care … complementary to clinical judgment and person-centered care”.

### 3.2. Relationship Between Compassionate Care and Peripheral Concepts

To make sense of variations in the relationships between the central concept of compassionate care and the peripheral concepts shown in the FCM metamodel, results regarding the centrality values (the sum of indegree and outdegree relationships) are shown in Table 2 below. While the centrality value as a quantitative measure does not distinguish between positive and negative influence, qualitative data from the deliberative discussions clarified participant perceptions of the relationships.

**Figure 2:**
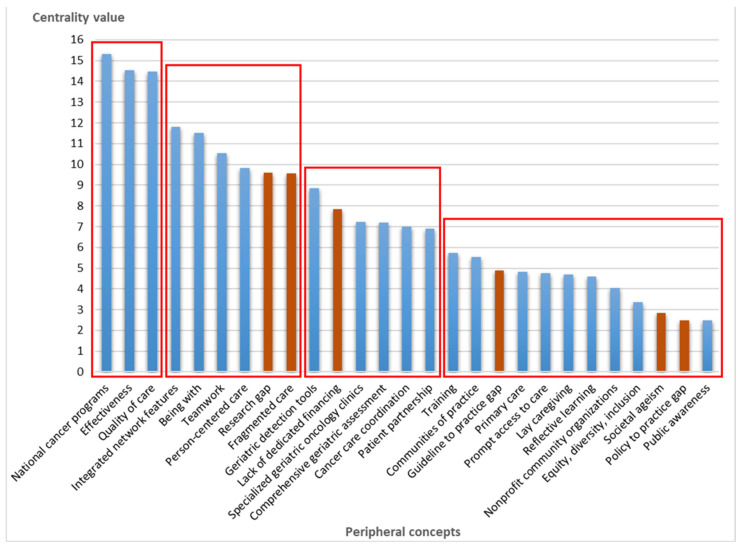
Red boxes illustrate clusters of peripheral concepts related to compassionate care of older adults with cancer. Blue bars illustrate concepts with a positively perceived relationship. Orange bars illustrate concepts with a negatively perceived relationship.

Figure 2 presents four clusters resulting from the semi-quantitative clustering approach to understanding concepts and extracting practical guidance from results.

The concept “national cancer programs” has the highest centrality value among peripheral concepts (C = 15.32), followed by “effectiveness” (C = 14.53) and “quality of care” (C = 14.48). This first cluster in Figure 2 reflects the determinants at a macro decision-making level. The participants considered that national cancer programs had enabled the steady development of clinical care and service organizations along with a person-centered approach over the past two decades. Discussion during the focus groups highlighted the priority orientations of the Québec Cancer Program that, while not mentioning older adults specifically, include the active participation of people living with cancer, continuous learning, and practice improvement. France’s program, issued by the French National Cancer Institute, emphasizes structured geriatric oncology and provides specific recommendations for older adults. Participants acknowledged that local initiatives bring compassion-driven improvements but feel these are insufficient to ensure resources and sustainability. The deliberation led to this perception: “Local initiatives are very important. Everyone believes in compassion, and then everyone in their community tries to do what’s best for patients. But small local initiatives have a certain limit in terms of resources resulting in very heterogeneous care delivery from one community to the next. As a result, not everyone has equal access. So, it’s clear that we really need a geriatric oncology component that comes from the national cancer programs”. Québec participants also raised concerns about the potential impact of structural reforms that could reduce the resources allocated to cancer care, especially as they are not embedded as a legal obligation as they are in France. Participants from France were optimistic that stronger alignment in a national strategy could support equitable access to appropriate care for older adults. This reflection reinforces the need for a formal geriatric oncology framework within broader cancer programs.

The second cluster includes four positively perceived concepts related to care delivery models comprising “integrated network features”, “being with”, “teamwork”, and “person-centered care”, which each have centrality values ranging from 11.81 to 9.82. Participants described “being with” as relational proximity between professionals and patients throughout the care trajectory and related it to person-centered care. A participant stated that “I may be an 81-year-old man with cancer, but I am first and foremost a person… I’m not going to fight cancer; I’m going to live as fully as possible”. Compassionate teamwork involves putting the patient’s needs, values, and preferences front and center and considering that “care IS a relationship with an emphasis on reciprocity between care providers and users supported by integrated models of care”.

The third cluster includes five positively perceived concepts related to a geriatric approach (“specialized geriatric oncology clinics”, “geriatric detection tools”, “comprehensive geriatric assessment”, “cancer care coordination”, “patient partnership”), where centrality values range from 8.86 to 6.90. These second and third clusters relate to meso- (organizational) and micro- (clinical) levels: “The providers who do the evaluation don’t really see the global impact on the patient or the time invested by other care providers. Whereas if providers were connected as a team to create compassionate personalized care for our elders, well, we would see a different dynamic”.

The fourth cluster includes concepts with centrality values ranging from 5.74 to 2.49 and relates to the outer context of care delivery, institutions, and society. Even if the scope of centrality is greater than the other clusters, the qualitative data invite us to group these concepts into a single cluster. Participants identified knowledge and networking as central to optimizing a compassionate geriatric approach in oncology. They emphasized “training in geriatrics and compassion”, “communities of practice” to exchange learning and best practices, and “primary care and nonprofit community organizations” to support whole-person care along the cancer journey. Participants further stressed the need for ongoing support in the workplace to model and encourage collaborative approaches. Participants acknowledged that compassion was sometimes lacking in their healthcare system and considered that this reflected how ageing is regarded in society at large: “We [as a society] need to change the way we look at things, learn to tame ageing and detect frailties, co-create senior-friendly municipalities and combat ageism”. Integrating nonprofit community organizations to bridge specialized cancer care and home life would be helpful and would support self-management and lay caregivers. Older adults often have to be encouraged to use community resources: “We need to change the way seniors look at themselves; sometimes they feel old, useless, or that they’re not worth the bother”. A somewhat low centrality value was found for professional education and training: “The initial training of professionals ignores care delivery for this clientele”. Participants mentioned the limited number of specialists in geriatric oncology, and a failure to systematically include geriatricians in cancer care pathways and link them with oncologists and family physicians.

Globally, 6 out of the 27 concepts were perceived to have a negative relationship with compassionate care for older adults with cancer. The concept “research gap” has a relatively high centrality value (C = 9.61), reflecting “that older adults are less often recruited in clinical and intervention research, impeding best practices and effectiveness of care”. Participants pointed not only to the limited inclusion of older adults in cancer treatment but also to a lack of research aimed specifically at better understanding tailored interventions for older adults. The high centrality of “fragmented care” (C = 9.56) suggests uncertainty around what models would best enable a compassionate geriatric approach in oncology: “We are still facing an unanswered question: create dedicated geriatric oncology clinics or ensure that a geriatric approach is available to those who need it”. The concept of a “lack of dedicated financing” (C = 7.85) for geriatric oncology was also mentioned: “Small, local initiatives like access to specialized resources rest on individual leadership and have a limit in terms of financing… They are very heterogeneous from one environment to another”. The concept of a “guideline to practice gap” (C = 4.9), reflecting a lack of awareness and uptake of the existing practice guidelines, was thought by some participants “to be related to the opacity of the guideline development process or to their maladaptation to practice contexts”. Others argued that “guidelines have existed for many years but implementation lags behind, possibly due not only to lack of awareness, but also to the scarcity of professional resources always targeted in budget constraints”. Finally, the concepts “societal ageism” (C = 2.83) and “policy to practice gap” (C = 2.5) represent the larger environment of cancer care. Participants considered that “societal views and general policies on ageing compromise ‘compassionate caring’”. They saw a need for policies to better support lay caregivers (frequently older adults themselves), coordinate with nonprofit community organizations, and assure equity. Participants felt they had fewer levers to act on public perspectives on older people that might require social marketing and public awareness campaigns. The need to connect policies on ageing and cancer care programs was also mentioned.

## 4. Discussion

The first objective of this study was to deepen our understanding of the relationship between the concepts involved in implementing a compassionate geriatric approach in oncology that integrates evidence-based priorities for improvement. The participants shared different ways that compassionate care is enacted in current geriatric oncology but collectively positioned compassionate care as an intrinsic component of high-quality and responsive care for older adults with cancer [20]. Shedding light on the subjective and relational nature of concepts such as compassionate care provides a starting point to improve its integration as a necessity [22,23,59]. As for our second objective, focusing on different stakeholder views around the positive or negative influence of various concepts, participants shared a diversity of representations that linked multiple enablers and barriers to a compassionate approach in geriatric oncology. The centrality values from cognitive mapping reflect opportunities that may help draw up priority orientations and actions for improvement to better integrate a compassionate geriatric approach in oncology, as per our third objective. The following sections discuss the multi-level determinants of compassionate care as key assets to reconcile geriatric oncology from the perspective of patients, lay and professional care providers, researchers, and organizational and policy decision-makers.

Our study identified relationships between the concepts relevant to translating established evidence-based priorities into compassionate geriatric oncology care. The results provide original information from different stakeholders (patients, lay and professional care providers, researchers, and organizational and policy decision-makers) from two health systems. None of the predetermined concepts were left out, which suggests that they all exert a positive or negative influence on compassionate care, although the relationships between some concepts are much stronger than others.

The results underline that compassionate care requires work at multiple levels from macrosystem governance (national programs), to organizational support, to care delivery models, and outward to society to combat ageism [20,25,60,61]. Based on the literature and considering the complex relationships between concepts in the metamodel, the following sections describe how these results can contribute to drawing up realistic priority orientations and actions for improvement towards compassionate care for older adults with cancer at each level.

### 4.1. Promoting Compassionate Care at the Systemic Level

Participants saw cancer programs that set national objectives for effective high-quality care as having the highest central value [62]. This suggests a call to action at the governance level to connect policies on ageing and cancer care programs [63] and include provisions on cancer care for older adults in these programs [14].

Québec’s national cancer program [64] aligns with international priorities including the active participation of people living with and beyond cancer, continuous learning and practice improvement, appropriate treatment regimens, and support across the care trajectory [3,11,14]. Participants expressed uncertainty about the impact of reforms, recognizing that these play a key role in shaping cancer care policies. France’s cancer program specifically addresses geriatric oncology and the specific needs of older adults [65]. There are concerns about upcoming financing reforms that, while potentially enhancing primary prevention strategies, may also reduce funding for cancer screening and regional access to care. These results underline the importance of policy frameworks to support ongoing efforts to improve practice at a local level. While improvements were seen in screening and treatment guidelines [66], participants called for a greater emphasis on policies at the macro-level and real-world conditions to globally address the challenges of compassionate cancer care in ageing populations [67].

The challenge of translating priority orientations into compassionate care rests on the mobilization and coordination of collective capacities across multiple providers that address the whole person and their unique trajectory. Inequities are evident across the continuum [3]. Different approaches have been attempted in various jurisdictions, but significant gaps persist. Older adults with cancer contend with the gaps, barriers, and challenges to obtaining care [68]. Our results strongly suggest that a mobilization of governance at the national level is crucial to address the long-standing deficiencies in service provision for this growing clientele. An overarching model of compassionate care delivery based on normative integration of evidence-based priorities and functional integration of real-world practice contexts could strengthen system resilience to face the “grey tsunami” in cancer that is now upon us. However, even with an explicit commitment at the policy level and substantial efforts from healthcare professionals, widespread access to integrated geriatric oncology is constrained by human resource challenges. With a scarcity of resources and a lack of multi-level commitment and support, professional and lay care providers are at risk of compassion fatigue [21,61].

National cancer programs can incorporate input from older adults and promote mechanisms for top-down and bottom-up connections to embed compassionate care as a central component of effectiveness and quality [61]. Our results (including the relatively high centrality of integrated network features [69]) support the promotion of integrated cancer networks to avoid gaps in cancer care and support self-management in older adults. Nurturing and sustaining compassionate care for older adults with cancer requires a common cognitive model, meaning a shared vision, values, and objectives between organizations, professionals, lay caregivers, and older adults with cancer. Collaborative governance is a promising approach that drives two-way dynamics at macro-, meso- and micro-levels in joint action sustaining collaboration within and between healthcare teams, and multisystem integration [70]. The fight against fragmentation may be better achieved with a collaborative governance approach that sees policy-level mobilization as one part of the solution alongside network-based practices involving managers, care providers, and partnerships with services users.

### 4.2. Integrating Compassionate Care at the Organizational Level

The fragmentation of care is perceived by the participants as the organizational factor most associated with impeding compassionate care. Fragmentation works against positive factors such as organizational proximity, coordination, and teamwork [12,13] and leads to inequity and adverse health impacts [68]. More than 10 years ago, a literature review on integrated geriatric approaches in oncology found that most research focused on the development and use of geriatric assessments, while very little explored care coordination, and none addressed specific integrated approaches to care for older adults [13]. Recent studies still call for work on the organizational and practical changes, and on approaches that provide direction for multi-level interventions at the clinical, organizational, and strategic levels [3,10,29]. Understanding the mechanisms that enable effective interdisciplinary teamwork is a first step to reducing fragmentation and achieving anticipated outcomes [71]. Mechanisms operate at multiple levels from cancer networks at the systemic level to service delivery settings [70]. At the team level, the intensity of interdisciplinary teamwork influences patient experience of care, and perceptions of access, continuity, communication, and person-centeredness [21,72,73].

Longer survival and follow-up periods have heightened the importance of primary care providers to people affected by cancer [74]. Collaboration and transitions between oncology and primary care teams have proven problematic [12], and despite their potential value, the adoption of risk-stratified models of follow-up care for cancer survivors has been slow [52]. The participation of people living with cancer as partners at organizational and policy levels [64,75] is a means of ensuring that decisions integrate both scientific and experiential knowledge and consider the impact decisions will have on patients [76,77].

These considerations relate to the debate around how best to organize geriatric oncology care, whether dedicated clinics are needed, and who should be involved. A recent study of clinics in Canada and the United States found a variety of structures, staffing models, and referral patterns [78]. Most were run by either geriatric oncologists or by geriatricians and oncologists, and had nursing, social work, and pharmacy services. Clinicians with training in both geriatrics and oncology remain rare. Geriatric assessment was used but, similarly to the findings in a study by Lafaie and colleagues, the tools employed and professionals responsible for an assessment varied significantly [79]. While there have been calls to standardize geriatric assessments in oncology, some authors consider that broad consensus on any one choice would be impossible to achieve [48]. They also stress the primacy of clinical judgment: scores can predict some adverse outcomes but “have less meaning if we do not interpret them within the context of the patient” [48] (p. 2). This view stresses the disparities between considerations regarding key factors in chemotherapy treatment and geriatric autonomy by oncologists and geriatricians. The priority placed on relational proximity in compassionate care we found in this study aligns with the importance of reconciling the perspectives of medical specialists with personalized care [79].

### 4.3. Translating Compassionate Care into Real-World Practice

Translating compassionate cancer care into practice appears complex and challenging. Practice-level factors include both professional values such as person-centered care [80], and the use of geriatric assessment tools and practice guidelines. Assessments of the values and preferences of older patients with cancer are critical to inform treatment decision-making. Is the patient willing to consider chemotherapy? Is the patient willing to accept treatment-related toxicities in exchange for the potential survival benefit a treatment affords? How important to the patient is maintaining quality of life and functional independence during treatment?

A compassionate response to the suffering and needs of an older adult with cancer requires relational understanding, reciprocity, and shared objectives between care providers and users [20,25,61]. From a patient perspective, this response includes feeling cared for, sensitivity, kindness, understanding needs, and a “warm presence” [81]. Older adults’ conceptions of geriatric oncology involve providers “being with” them in their decision-making [82] and considering them as full members of interdisciplinary teams across care settings [73]. This relational proximity between provider and patient expands to person-centered care in which older adults with cancer and/or their relatives are involved in organizational committees. This ensures that activated patients, lay caregivers, and prepared proactive healthcare teams embrace productive, compassionate relationships.

### 4.4. Bridging Health Systems and Societal Context

The results reveal the importance of the global context at a societal level to compassionate care, although the concept was deemed less central by study participants. The onus on equity, diversity, and inclusion requires adapting the cancer research agenda. This calls for better representation of the older and oldest adults affected by geriatric syndromes and comorbidities, along with research into interventions to optimize the health of older patients with cancer [10,56]. The dearth of research impedes the uptake of practice guidelines, and older adults are often excluded from clinical trials. However, there are efforts in some countries to widen eligibility and limit participation only to people with the most severe comorbidities [10]. Some authors suggest doing away with randomization and designing trials that allow a patient and their healthcare team members to select a treatment option [10].

The documented gaps in initial training and continuing education programs for professionals worldwide hinder progress [83]. Geriatric oncology training is essential to ensure that professionals develop their competency in caring for older adults with cancer [78]. France has deployed advanced training in geriatrics for healthcare professionals in a number of dedicated geriatric oncology clinics to enable mentorship. This aligns with the views that exposure to geriatric medicine during training for all medical, nursing, and allied health professionals is critical [84].

Our findings reflect the need to address ageism, which remains under-recognized in cancer care [84]. Our results help to understand ageism as a barrier to catalyzing health system development to face the acceleration of the ageing population and the increasing number of older adults with cancer that will accompany this demographic shift. Ageism has been flagged as insidious in cancer care for decades [85]. A recent worrying report in Canada reveals the following concerns: older adults being ignored or treated paternalistically in healthcare systems; healthcare providers assuming that symptoms are “just due to age”; and older adults being denied care or viewed as disposable, unworthy, and a burden on a healthcare system [86]. Among clinicians, ageism in clinical decisions may arise from well-meaning intentions to consider differences in age-related biology that could influence treatment effectiveness and outcome [87]. Patient-centered compassionate care must guard against age stereotypes that increase the risk of so-called compassionate ageism [88].

Unfortunately, the increase in the number of older adults living with cancer has not been mirrored by a proportional increase in investments in the health services required to respond to the unique needs of this group. Nor has there been sufficient attention to organizing services to meet their needs in different contexts, from specialized care to nonprofit community organizations. At the practice level, a lack of investment in producing evidence specific to older adults with cancer contributes to clinical uncertainty [7]. At the policy level, support for initiatives, often of community organizations, are insufficient to meet older adults’ social needs, whether transport or navigation assistance or psychological support [8,9], and negatively impact their ability to manage cancer and its treatment. While pivot nurses were introduced into Québec’s cancer program early on, budgetary and human resource shortages have jeopardized their availability to coordinate care within and between teams [70]. Integrated care is regularly mentioned as a policy priority; however, inadequate support from healthcare systems or policies along with poor linkage between disciplines and healthcare services perpetuate fragmented care for older adults with cancer [12]. Finally, public perceptions on ageing are seen as exerting a negative influence on providing personalized care for older adults with cancer.

### 4.5. Implications

There is no “typical older adult”, nor generalizable protocol, only recognition that older adults may be more vulnerable to treatment toxicities that can affect all aspects of their life [2,15]. This suggests that a geriatric approach in oncology involves evaluating a person’s medical, physical, emotional, and informational needs, and understanding the concerns older adults with cancer and their lay caregivers have around health, quality of life, or end of life [89]. The concept of compassionate care, addressed as a complex intervention, appears key to catalyzing action on the longstanding call to improve care for older adults. Currently, organizational readiness and capacity to change practices mostly rest on individual leadership while improvements in training, management, and communication require multi-level mobilization. This study reveals the importance of providing relevant policies, structures, and dedicated resources to support the translation of compassionate care into practice. The findings on stakeholder perspectives of compassionate care and the difficulties of providing it inform a crucial first step in evaluating a sustainable implementation of compassionate approaches. Future research using an implementation science framework such as RE-AIM could guide context-specific adaptation [90] and help reduce the gap between priority orientations, policy, and practice.

The ability to capture relationships, handle multiple variables, and incorporate human expertise has made FCM valuable in various fields, including economics, finance, engineering, and social sciences. The use of FCM in medicine and health care is recent but is recognized as a powerful tool for modelling complex systems, particularly those characterized by nonlinear dynamics, uncertainty, and multiple interacting elements [38]. Creating an FCM involves a structured and iterative process (Figure 1) to represent dynamic relationships within complex systems, enabling proactive research to advance the utility and integration of knowledge issued from exchanges between multiple stakeholders.

### 4.6. Strengths and Limitations

To the best of our knowledge, this study is the first to capture multi-stakeholder deliberation on compassionate care in geriatric oncology. FCM is gaining recognition as an interpretable and transparent process to address complex problems [91], producing results that can inform interventions and action plans. The participants easily engaged in the deliberative process and expressed their appreciation for the rich exchanges between care providers and users. Some participants then volunteered to take part in the two member checking sessions, which increased the transparency and the credibility of the results [58]. Assembling different perspectives into one shared mental model represents a step forward to activating compassionate geriatric care in oncology. A particular strength of this study is the inclusion of multiple perspectives in our groups that revealed a strong consensus around the importance of addressing barriers to compassionate care in geriatric oncology to reduce the burden of unnecessary suffering. Facilitator preparation and prior experience in focus group research helped manage the biases related to power dynamics in the multi-stakeholder groups. From a methodological perspective, the FCM undertaken in this study adds an empirical basis to the prior utilization of FCM in health domains [37,38]. The validity relies on our systematic process, expert participants, and member checking.

This study has some limitations. First, non-probabilistic sampling may have introduced biases, as the participants had expertise and held strong views about challenges in geriatric oncology and patients’ need for compassion. Deliberations with different stakeholders in other contexts could have produced FCM results showing lower centrality for “compassionate care”, with linkages being made primarily between peripheral concepts, and fewer between peripheral concepts and compassionate care. Second, while the use of predetermined concepts was helpful in focusing the discussion considering the time (90 min) that could reasonably be allotted to mapping, it may have limited the variability of participant perspectives. A third limitation relates to the challenge of making pragmatic sense of FCM while integrating both quantitative centrality values and qualitative perceived positive and negative influences. We used a clustering approach in our interpretation to contend with the large number of enmeshed and multidirectional relationships between peripheral concepts that nevertheless reflect realities in the practice environment. This effort to manage complexity entails some risk of over-simplification. However, the integration of complementary qualitative and quantitative data, supported by member checking from people with lived experience and scientific knowledge, contributes to internal validity [58].

## 5. Conclusions

This study produces new information on the intricate relationships between concepts that enable or constrain the translation of scientific evidence and established priorities into compassionate geriatric oncology care. Cognitive mapping shows it is possible to arrive at a shared understanding of complex problems that integrates the cognitive representations of multiple stakeholders. This effort provides a basis for progressing to a participatory co-design and evaluation of interventions in context. Our results underline, once again, that work is needed across organizational and professional boundaries and at policy, organizational, and clinical decision-making levels. Understanding the complexity of acting on the established priorities appears to be key to overcoming longstanding gaps in the care of older adults with cancer.

## Figures and Tables

**Table 1:** Participant characteristics (total *n* = 53).

Characteristics	Mean (SD)	Frequency	%
Age (years)	48.29 (12.74)		
Gender			
Woman		45	84.91
Man		8	15.09
Country			
Canada		47	88.68
France ^1^		6	11.32
Function ^2^			
Allied health professional ^3^		25	47.17
Oncologist		5	9.43
Geriatrician		4	7.55
Manager		9	16.98
Director		8	15.09
Researcher		13	24.53
Policymaker		8	15.09
Older adult with cancer ^4^		5	9.43
Experience in oncology (years)			
Less than 3		16	30.19
3 to 5		6	11.32
5 to 10		11	20.75
More than 10		20	37.74
Institutional affiliation ^5^			
Health setting—academic mandate		14	26.42
Health setting—community mandate		28	52.83
Government ^6^		8	15.09
University		4	7.55
Other		1	1.89

^1^ One participant had an international perspective. ^2^ Some participants occupied more than one function and were affiliated with more than one institution. ^3^ Allied health professionals: nurses, social workers, pharmacists, occupational therapists. ^4^ Older adults with cancer: patients and their caregivers. ^5^ Affiliation or habitual source of care for older adults with cancer. ^6^ Government: provincial ministry or municipal level.

**Table 2:** Centrality and perceived relationship of peripheral concepts with compassionate care of older adults with cancer based on FCM metamodel.

	Centrality (C) ^1^	Indegree	Outdegree	Perceived Relationship
Central concept				
Compassionate care of older adults with cancer	19.71	19.71	0.00	---
Peripheral concepts				
National cancer programs	15.32	8.00	7.32	Positive
Effectiveness	14.53	9.72	4.81	Positive
Quality of care	14.48	9.51	4.97	Positive
Integrated network features	11.81	6.31	5.50	Positive
Being with	11.50	5.00	6.50	Positive
Teamwork	10.55	6.35	4.20	Positive
Person-centered care	9.82	5.61	4.21	Positive
Research gap	9.61	4.36	5.25	Negative
Fragmented care	9.56	6.75	2.81	Negative
Geriatric detection tools	8.86	3.25	5.61	Positive
Lack of dedicated financing	7.85	2.50	5.35	Negative
Specialized geriatric oncology clinics	7.23	0.70	6.53	Positive
Comprehensive geriatric assessment	7.19	2.28	4.91	Positive
Cancer care coordination	7.01	3.31	3.70	Positive
Patient partnership	6.90	1.40	5.50	Positive
Training	5.74	0.45	5.29	Positive
Communities of practice	5.55	1.20	4.35	Positive
Guideline to practice gap	4.90	1.58	3.32	Negative
Primary care	4.81	1.31	3.50	Positive
Prompt access to care	4.75	2.25	2.50	Positive
Lay caregiving	4.68	2.80	1.88	Positive
Reflective learning	4.60	2.47	2.13	Positive
Nonprofit community organizations	4.03	0.78	3.25	Positive
Equity, diversity, inclusion	3.37	1.00	2.37	Positive
Societal ageism	2.83	1.00	1.83	Negative
Policy to practice gap	2.50	0.74	1.76	Negative
Public awareness	2.49	0.75	1.74	Positive

^1^ Centrality is based on absolute weight values; it is always a positive number. Perceived positive or negative relationship relates to the qualitative description from participants.

## Data Availability

The data presented in this study are available from the corresponding author upon reasonable request.

## Supplementary Materials

The following supporting information can be downloaded at https://www.mdpi.com/article/10.3390/curroncol32080465/s1: Table S1: Definitions of concepts. Figure S1: Metamodel aggregating maps from eight focus groups. References [3,11,14,15,20,21,26,41,48,49,50,51,62,63,67,68,69,71,72,74,77,80,81,82,84] are cited in the Supplementary Materials.

## Abbreviations

The following abbreviations are used in this manuscript:

C	Centrality
FCM	Fuzzy cognitive mapping
PRISM	Practical robust implementation and sustainability model
RE-AIM	Reach, effectiveness, adoption, implementation, maintenance
SIOG	International Society of Geriatric Oncology
