# Supplementary material for: Exploring Determinants of Compassionate Cancer Care in Older Adults Using Fuzzy Cognitive Mapping
Source: Curr Oncol. 2025 Aug 16;32(8):465. doi: 10.3390/curroncol32080465 (PMC12385172; doi:10.3390/curroncol32080465)

Supplementary Figure S1. Metamodel aggregating maps from eight focus groups.

This figure illustrates the aggregation of fuzzy cognitive mapping (metamodel) from eight deliberative focus groups. It shows the entanglement of relationships between compassionate care (central concept) and 27 peripheral concepts. Blue lines show positive and orange lines negative relationships between concepts. Black dots show relationships based on aggregated weight values. This is the output from Mental Modeler software.

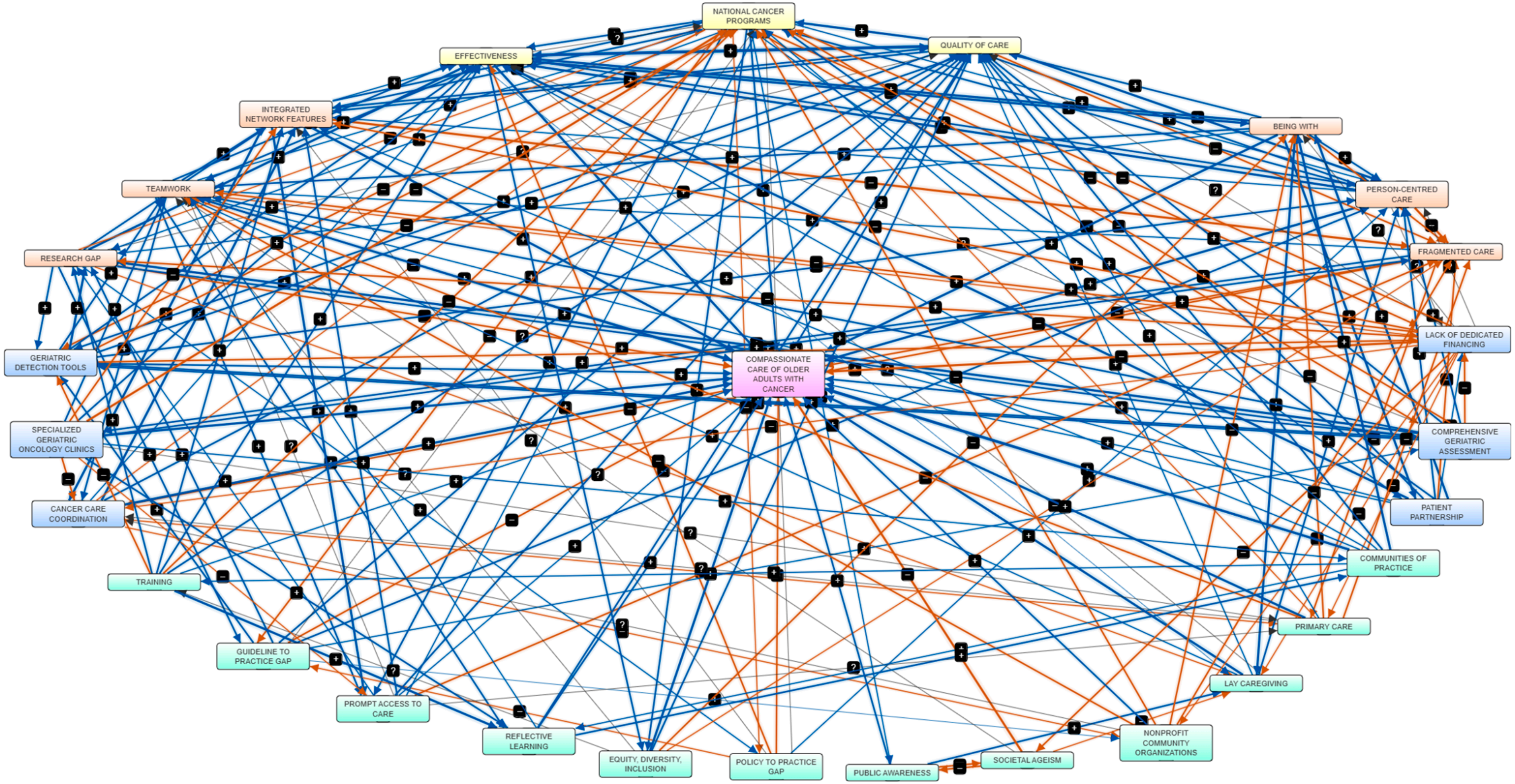

Supplement: Supplementary file 1 [file curroncol-32-00465-s001.zip › curroncol-3715968-Supplementary_Figure S1.pdf]
