# Supplementary material for: Exploring Determinants of Compassionate Cancer Care in Older Adults Using Fuzzy Cognitive Mapping
Source: Curr Oncol. 2025 Aug 16;32(8):465. doi: 10.3390/curroncol32080465 (PMC12385172; doi:10.3390/curroncol32080465)
Supplement: Supplementary file 1 [file curroncol-32-00465-s001.zip › curroncol-3715968-Supplementary_Table S1.pdf]

Current Oncology
Exploring Determinants of Compassionate Cancer Care in Older Adults Using Fuzzy Cognitive Mapping
Tremblay D. et al. 2025

Supplementary Table S1. Definitions of concepts.

The following lexicon provides definitions of concepts presented to participants for the fuzzy concept mapping (FCM) session. Definitions applied in this FCM were adapted from scientific literature and empirical research.

Concepts	Definitions applied in this FCM
Being with	Personal commitment to the relationship with an older adult with cancer, which manifests, among healthcare professionals and institutional staff, as a genuine interest in how the person is doing, and in support and regular communication with lay caregivers that respects their complementary role [1].
Cancer care coordination	Organization of and connections within and between the services and information a person needs to obtain care and support throughout their cancer care trajectory [2].
Communities of practice	Forums that enable groups of professionals with a common set of concerns or problems to develop and share knowledge and expertise through ongoing interactions [3].
Compassionate care of older adults with cancer	A caring relationship where providers are concerned about and proactively attend to a person's suffering and needs [4-7].
Comprehensive geriatric assessment (CGA)	Assessment of a person's more general medical, functional and psychosocial health to identify vulnerabilities in addition to those related to cancer that may influence care decisions [8,9].
Effectiveness	Ability to produce the outcomes intended by an intervention in health care [10].
Equity, diversity, inclusion	Providing fair and just treatment to older adults, regardless of their age, sex, gender, ethnicity, language, place of residence or socio-economic status [11,12].
Fragmented care	Lack of connection and coordination between care received from providers in different places and over time [13,14].
Geriatric detection tools	Validated screening tools used to evaluate vulnerabilities in older adults with cancer (e.g., Geriatric-8 (G8) and Vulnerable Elders Survey-13 (VES-13) [15].
Guideline to practice gap	Challenges in providing care that corresponds to clinical practice guidelines, which are evidence-based recommendations for optimal cancer care in older adults [16,17].
Integrated network features	Structures and processes at clinical, organizational and system level that work to produce relationships and coordinate interdependencies among actors, including specialized cancer care, primary care and nonprofit organizations [18].
Lack of dedicated financing	Lack of investment, funding and financial incentives for the development of geriatric oncology services, professional development and training, and basic, clinical and translational multidisciplinary research to improve the care of older adults with cancer [16,17].
Lay caregiving	Ongoing care provided by a relative, friend or partner who has a significant relationship with an older adult with cancer and assists with that person’s medical care, personal care, household tasks, and social and emotional needs [19].
National cancer programs	Public health programs that provide plans and evidence-based strategies for cancer prevention and control across a country or state/province and aim to improve cancer patients’ quality of life [20].

Concepts	Definitions applied in this FCM
Nonprofit community organizations	Community organizations or local chapters of philanthropic organizations with a charitable mission providing services that meet the needs of cancer patients and lay caregivers, in complementarity to those offered by cancer care institutions [21,22].
Patient partnership	Role based on experiential knowledge and expertise played by people living with or beyond cancer to support others along the continuum of care and services (e.g., as lay navigators), and to advise on the evaluation and improvement of care quality and the organization of care and services (e.g., on national committees). At governance level, patient partners contribute their perspective at all levels, from micro (service delivery) to macro (strategic planning) [23].
Person-centered care	Care where a person's particular situation, needs, preferences and abilities are considered in a shared decision-making process that also recognizes the role of lay caregivers [24].
Policy to practice gap	Challenges in translating government programs that set strategic direction for care and services to older adults into practice [25].
Primary care	The initial point of contact a person has with the healthcare system, where a family physician supported by a multidisciplinary team provides ongoing care [26].
Prompt access to care	Access to care without long wait times, within a reasonable distance, and at convenient times [27].
Public awareness	General population’s knowledge and understanding of subjects such as healthy aging, age-appropriate care, the occurrence of cancer and other diseases, and cancer care quality issues [17].
Quality of care	The extent to which healthcare services are evidence-based, contribute to achieving expected health outcomes, and are provided competently and respectfully [28].
Reflective learning	The effort of drawing lessons from experience by reflecting on beliefs and assumptions that shape one's perceptions and actions, and applying learnings toward improvement [29].
Research gap	Lack of research, including clinical trials, translational research, intervention research, and meta-analyses, to advance scientific knowledge related to geriatric oncology [17,30].
Societal ageism	Societal views on aging and older people that are reflected in stereotypes, prejudice and/or discrimination based on a person's age [31].
Specialized geriatric oncology clinics	Multidisciplinary clinics specialized in geriatric oncology that provide access to comprehensive geriatric assessment and include healthcare professionals who support oncology teams in addressing older patients’ needs and providing recommendations for optimal treatment plans [32].
Teamwork	Behaviors, knowledge and attitudes involved in working together interdependently [33].
Training	Initial and continuing education on evidence-based practices aimed at developing the knowledge and skills of health professionals, including compassion education [17,34].

References

1. Joannette, S. Signification Accordée à l'Approche Oncogériatrique Intégrée par des Personnes Agées Atteintes de Cancer. Master diss., Université de Sherbrooke, Longueuil, CAN, 2016. Available online: http://hdl.handle.net/11143/8190 (accessed on 5 March 2025).

2. Chollette, V.; Weaver, S.J.; Huang, G.; Tsakraklides, S.; Tu, S.-P. Identifying cancer care team competencies to improve care coordination in multiteam systems: A modified Delphi study. *JCO Oncol. Pract.* **2020**, *16*, e1324-e1331, doi:10.1200/op.20.00001.

3. Wenger, E.; McDermott, R.A.; Snyder, W. *Cultivating Communities of Practice: A Guide to Managing Knowledge*; Harvard Business School Press: Boston, USA, 2002; p. 284.

4. Gilbert, P.; Catarino, F.; Duarte, C.; Matos, M.; Kolts, R.; Stubbs, J.; Ceresatto, L.; Duarte, J.; Pinto-Gouveia, J.; Basran, J. The development of compassionate engagement and action scales for self and others. *J. Compassionate Health Care* **2017**, *4*, 1-24, doi:10.1186/s40639-017-0033-3.

5. Malenfant, S.; Jaggi, P.; Hayden, K.A.; Sinclair, S. Compassion in healthcare: An updated scoping review of the literature. *BMC Palliat. Care* **2022**, *21*, 1-28, doi:10.1186/s12904-022-00942-3.

6. Habib, M.; Korman, M.B.; Aliasi-Sinai, L.; den Otter-Moore, S.; Conn, L.G.; Murray, A.; Jacobson, M.C.; Enepekides, D.; Higgins, K.; Ellis, J. Understanding compassionate care from the patient perspective: Highlighting the experience of head and neck cancer care. *Can. Oncol. Nurs. J.* **2023**, *33*, 74-86, doi:10.5737/2368807633174.

7. Sinclair, S.; Hack, T.F.; MacInnis, C.C.; Jaggi, P.; Boss, H.; McClement, S.; Sinnarajah, A.; Thompson, G. Development and validation of a patient-reported measure of compassion in healthcare: The Sinclair Compassion Questionnaire (SCQ). *BMJ Open* **2021**, *11*, e045988, doi:10.1136/bmjopen-2020-045988.

8. Mohile, S.G.; Dale, W.; Somerfield, M.R.; Schonberg, M.A.; Boyd, C.M.; Burhenn, P.S.; Canin, B.; Cohen, H.J.; Holmes, H.M.; Hopkins, J.O.; et al. Practical assessment and management of vulnerabilities in older patients receiving chemotherapy: ASCO guideline for geriatric oncology. *J. Clin. Oncol.* **2018**, *36*, 2326-2347, doi:10.1200/jco.2018.78.8687.

9. Seghers, P.A.L.; Alibhai, S.M.H.; Battisti, N.M.L.; Kanesvaran, R.; Extermann, M.; O'Donovan, A.; Pilleron, S.; Mislang, A.R.; Musolino, N.; Cheung, K.-L.; et al. Geriatric assessment for older people with cancer: Policy recommendations. *Glob. Health Res. Policy* **2023**, *8*, 1-8, doi:10.1186/s41256-023-00323-0.

10. Burches, E.; Burches, M. Efficacy, effectiveness and efficiency in the health care: The need for an agreement to clarify its meaning. *Int. Arch. Public Health Community Med.* **2020**, *4*, 1-3, doi:10.23937/2643-4512/1710035.

11. Haase, K.R.; Sattar, S.; Pilleron, S.; Lambrechts, Y.; Hannan, M.; Navarrete, E.; Kantilal, K.; Newton, L.; Kantilal, K.; Jin, R.; et al. A scoping review of ageism towards older adults in cancer care. *J. Geriatr. Oncol.* **2023**, *14*, 1-28, doi:10.1016/j.jgo.2022.09.014.

12. Canadian Cancer Society. Advancing health equity through cancer information and support services: Report on communities that are underserved. Available online: https://cdn.cancer.ca/-/media/files/about-us/our-health-equity-work/underserved-communities-report_2023_en.pdf?rev=17ad3c41ed3b4b3abd99a33482b89d2a&hash=C8D25526630D310980C8B79DA73EB7D6 (accessed on 5 March 2025).

13. Ross, L.W.; Townsend, J.S.; Rohan, E.A. Still lost in transition? Perspectives of ongoing cancer survivorship care needs from comprehensive cancer control programs, survivors, and health care providers. *Int. J. Environ. Res. Public Health* **2022**, *19*, 1-15, doi:10.3390/ijerph19053037.

14. Molina, G.; Qadan, M. Addressing fragmentation of care requires strengthening of health systems and cross-institutional collaboration. *Cancer* **2019**, *125*, 3296-3298, doi:10.1002/cncr.32337.

15. Garcia, M.V.; Agar, M.R.; Soo, W.-K.; To, T.; Phillips, J.L. Screening tools for identifying older adults with cancer who may benefit from a geriatric assessment: A systematic review. *JAMA Oncol.* **2021**, *7*, 616-627, doi:10.1001/jamaoncol.2020.6736.

16. Cook, S.; Alibhai, S.; Mehta, R.; Savard, M.-F.; Mariano, C.; LeBlanc, D.; Desautels, D.; Pezo, R.; Zhu, X.; Gelmon, K.A.; et al. Improving care for older adults with cancer in Canada: A call to action. *Curr. Oncol.* **2024**, *31*, 3783-3797, doi:10.3390/curroncol31070279.

17. Extermann, M.; Brain, E.; Canin, B.; Cherian, M.N.; Cheung, K.-L.; de Glas, N.; Devi, B.; Hamaker, M.; Kanesvaran, R.; Karnakis, T. Priorities for the global advancement of care for older adults with cancer: An update of the International Society of Geriatric Oncology Priorities Initiative. *Lancet Oncol.* **2021**, *22*, e29-e36, doi:10.1016/S1470-2045(20)30473-3.

18. Tremblay, D.; Touati, N.; Roberge, D.; Breton, M.; Roch, G.; Denis, J.-L.; Candas, B.; Francoeur, D. Understanding cancer networks better to implement them more effectively: A mixed methods multi-case study. *Implement. Sci.* **2016**, *11*, 1-9, doi:10.1186/s13012-016-0404-8.

19. Adashek, J.J.; Subbiah, I.M. Caring for the caregiver: A systematic review characterising the experience of caregivers of older adults with advanced cancers. *ESMO Open* **2020**, *5*, e000862, doi:10.1136/esmoopen-2020-000862.

20. Union for International Cancer Control (UICC). What is an effective national cancer control plan? Available online: https://www.uicc.org/news/what-effective-national-cancer-control-plan (accessed on 5 March 2025).

21. Adebayo, O.W.; Salerno, J.P.; Francillon, V.; Williams, J.R. A systematic review of components of community-based organisation engagement. *Health Soc. Care Community* **2018**, *26*, e474-e484, doi:10.1111/hsc.12533.

22. Gentil, B.; Usher, S.; Loignon, C.; Tremblay, D. Opening doors to using nonprofit community-based services to better cope with breast cancer: A descriptive interpretive study of women's experience. *Science of Nursing and Health Practices* **2024**, *7*, 51-66, doi:10.62212/snahp-sips.122.

23. Bombard, Y.; Baker, G.R.; Orlando, E.; Fancott, C.; Bhatia, P.; Casalino, S.; Onate, K.; Denis, J.-L.; Pomey, M.-P. Engaging patients to improve quality of care: A systematic review. *Implement. Sci.* **2018**, *13*, 1-22, doi:10.1186/s13012-018-0784-z.

24. Ebrahimi, Z.; Patel, H.; Wijk, H.; Ekman, I.; Olaya-Contreras, P. A systematic review on implementation of person-centered care interventions for older people in out-of-hospital settings. *Geriatr. Nurs.* **2021**, *42*, 213-224, doi:10.1016/j.gerinurse.2020.08.004.

25. Hudson, B.; Hunter, D.; Peckham, S. Policy failure and the policy-implementation gap: Can policy support programs help? *Policy Des. Pract.* **2019**, *2*, 1-14, doi:10.1080/25741292.2018.1540378.

26. Rubin, G.; Berendsen, A.; Crawford, S.M.; Dommett, R.; Earle, C.; Emery, J.; Fahey, T.; Grassi, L.; Grunfeld, E.; Gupta, S.; et al. The expanding role of primary care in cancer control. *Lancet Oncol.* **2015**, *16*, 1231-1272, doi:10.1016/S1470-2045(15)00205-3.

27. Vélez, M.; Bain, T.; Rintjema, J.; Wilson, M.G. Rapid Synthesis: Identifying the Features and Impacts of Cancer-Care Networks on Enhancing Person-Centered Care and Access to Specialized Services. **2021**.

28. Institute of Medicine; National Research Council; National Cancer Policy Board. Defining and assessing quality cancer care. *Ensuring Quality Cancer Care* **1999**, 79-115.

29. Patel, K.M.; Metersky, K. Reflective practice in nursing: A concept analysis. *Int. J. Nurs. Knowl.* **2022**, *33*, 180-187, doi:10.1111/2047-3095.12350.

30. Rostoft, S.; Seghers, N.; Hamaker, M.E. Achieving harmony in oncological geriatric assessment - Should we agree on a best set of tools? *J. Geriatr. Oncol.* **2023**, *14*, 1-3, doi:10.1016/j.jgo.2023.101473.

31. Krasovitsky, M.; Porter, I.; Tuch, G. The impact of ageism in the care of older adults with cancer. *Curr. Opin. Support. Palliat. Care* **2023**, *17*, 8-14, doi:10.1097/spc.0000000000000629.

32. Menjak, I.B.; Campos, K.; Pasetka, M.; Budden, A.; Curle, E.; Gibson, L.; Szumacher, E.; Mehta, R. Implementation of a multi-disciplinary geriatric oncology clinic in Toronto, Canada. *Curr. Oncol.* **2025**, *32*, 1-15, doi:10.3390/curroncol32020089.

33. Verhoeven, D.C.; Chollette, V.; Lazzara, E.H.; Shuffler, M.L.; Osarogiagbon, R.U.; Weaver, S.J. The anatomy and physiology of teaming in cancer care delivery: A conceptual framework. *J. Natl. Cancer Inst.* **2020**, *113*, 360-370, doi:10.1093/jnci/djaa166.

34. Sinclair, S.; Kondejewski, J.; Jaggi, P.; Dennett, L.; Roze des Ordons, A.L.; Hack, T.F. What Is the state of compassion education? A systematic review of compassion training in health care. *Acad. Med.* **2021**, *96*, 1057-1070, doi:10.1097/acm.0000000000004114.
